# A Narrative Review of Recent Insights on Nerve Growth Factor Signaling in Physiological and Pathological Ovarian Processes in Mammals

**DOI:** 10.3390/biom16050699

**Published:** 2026-05-08

**Authors:** Massimo Aloisi, Gianna Rossi, Sandra Cecconi

**Affiliations:** Department of Life, Health and Environmental Sciences, University of L’Aquila, 67100 L’Aquila, Italy; massimo.aloisi@univaq.it (M.A.); gianna.rossi@univaq.it (G.R.)

**Keywords:** NGF, mammalian ovary, infertility, folliculogenesis, ovarian diseases

## Abstract

Nerve Growth Factor (NGF), a member of the neurotrophin family, is currently regarded as a key regulator of ovarian physiology beyond its well-known neurotrophic functions. The mammalian ovary is one of the most highly innervated peripheral organs. Increasing evidence indicates that NGF and its receptors, TrkA and p75NTR, are widely expressed in ovarian tissues. Through the activation of the PI3K/AKT, MAPK/ERK, and PLCγ signaling pathways, NGF influences granulosa cell proliferation, steroidogenesis, and ovulation. Physiological levels of NGF are essential for primordial follicle activation, FSH receptor expression, and effective bidirectional communication between oocytes and surrounding somatic cells. As a result, NGF also regulates oocyte maturation and developmental competence. The disruption of NGF signaling can lead to serious health issues. Both low and high levels of NGF negatively affect folliculogenesis and fertility. Elevated intraovarian NGF results in sympathetic over-innervation, altered steroid production, and polycystic ovarian features. In addition, increased NGF expression has been linked to endometriosis and ovarian cancer progression. Clinical studies further suggest that follicular NGF levels may serve as indicators of ovarian reserve and reproductive outcomes in assisted reproduction. This narrative review synthesizes the current knowledge on NGF roles in ovarian physiology and disease. It highlights NGF’ dual functions as a central regulator of follicular dynamics, and as a potential biomarker and therapeutic target for common reproductive system diseases.

## 1. Introduction

Mammalian ovaries are dynamic organs responsible not only for producing and releasing oocytes, but also for secreting hormones and many signaling molecules with autocrine and paracrine functions. Ovaries undergo significant changes from fetal life onward and exihibit a progressive age-dependent decline in terms of their follicular reserve that, in humans, culminates with menopause, marking the end of the reproductive period. Folliculogenesis is the complex, multi-step process by which oocytes develop within the surrounding ovarian somatic cells, from the primordial stage to the ovulation of mature eggs [1,2]. These functions depend on bidirectional signaling between germ and follicular cells and on the effective communication between the ovary and the brain via the hypothalamic–pituitary–ovarian (HPO) axis. Disruptions within this tightly regulated network can impair physiological ovarian functions, decrease the ovarian reserve, and contribute to pathological conditions, such as polycystic ovary syndrome (PCOS), premature ovarian failure, and anovulation [3].

**Figure 1:**
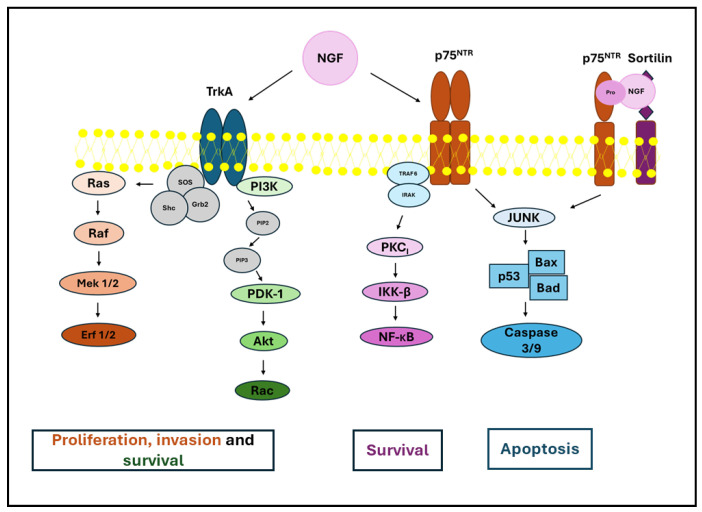
Pro-NGF and NGF-activated signaling pathways. Binding of NGF to TrkA receptor triggers multiple downstream cascades that promote cell survival and proliferation, while also potentially inducing apoptosis through p75NTR binding. The interaction between these signaling cascades is key in determining cell fate. For more details, see [12,13].

In recent decades, research has expanded beyond a solely endocrine viewpoint to emphasize the central role of the follicular microenvironment [4]. Consequently, greater focus has been placed on molecules once believed to be tissue-specific, such as Nerve Growth Factor (NGF). In fact, this growth factor is expressed in various non-neural tissues, including male and female reproductive organs, where it regulates numerous functions beyond its traditional neurotrophic role [5,6,7,8]. NGF is generated from the precursor pro-NGF after its proteolytic cleavage by matrix metalloproteinase 7 (MMP7), plasmin, and other proteases. Mature NGF preferentially binds to the trophic tyrosine kinase receptor TrkA, while pro-NGF binds to the neurotrophin receptor complex formed by p75NTR and sortilin. The two growth factors have distinct biological effects: while the binding of NGF to TrkA activates intracellular pathways usually regulating cell cycle progression and survival, such as PI3K/AKT, MAPK/ERK, and PLCγ [9,10], the activation of p75NTRby pro-NGF or NGF can determine different biological effects dependent on cellular context, but usually leads to cell death via the activation of JNK and caspase pathways [11,12,13] (Figure 1).

To write this review, we searched on PubMed and Scopus for research articles and reviews published until March 2026 using keywords such as “NGF and female fertility”, “NGF and ovaries”, and “NGF in health and disease”. We added comments and perspectives based on the reported results. The “narrative” approach is used to make the reading more fluid, as our first aim is to introduce the readers to an important topic that is still poorly investigated.

## 2. NGF and Receptors in the Mammalian Ovary

The mammalian ovary is one of the extra-neural tissues with the highest NGF expression. In this organ, the sympathetic and parasympathetic systems work together to ensure the stable regulation of the neuroendocrine ovarian axis. Catecholaminergic neuron-like cells are present in the ovarian cortex, closely associated with follicles, while the medulla contains a denser neurovascular network [14]. The importance of ovarian innervation is demonstrated by the significant inhibition of follicular growth observed after mechanical denervation [15].

The localization of NGF and its high-affinity receptor TrkA has been extensively studied in rat ovaries. During fetal development, NGF and receptor transcripts are highly expressed, then decline during neonatal and prepubertal stages, and rise sharply again at puberty, coinciding with the Luteinizing Hormone (LH) surge and ovulation [16]. Notably, NGF is the only neurotrophin reported to be upregulated during this ovulatory inflammatory-like process [17]. Experimental data from rat ovaries have shown that mRNAs for NGF, TrkA, and p75NTR are abundant in the theca cells (TCs) of antral follicles, with TrkA also present in granulosa cells (GCs) [18]. The ability of NGF to enhance follicular responsiveness to gonadotropins is supported by findings that in cultured neonatal rat ovaries, NGF stimulates the accumulation of FSH receptor (FSHR) mRNA to levels sufficient to promote the transition to the gonadotropin-dependent stage of follicular development and the release of Vascular Endothelial Growth Factor (VEGF) that stimulates ovarian vascularization [19].

In mice, NGF and TrkA are present in fetal ovaries before follicle assembly; at later stages, NGF is abundant in GCs, TCs, interstitial cells, and oocytes, as well as in follicular fluid (FF), whilst TrkA and p75NTR are primarily localized in the somatic compartment [20,21]. Although the absence of NGF postpones but does not prevent primordial follicle assembly, the transition from quiescent to the growing stage (i.e., follicle activation) is dramatically impaired [22,23].

In sheep ovaries, NGF has been detected in FF of antral follicles, while TrkA expression is restricted to cumulus cells (CCs) and GCs [24]. In pigs, neuronal cells and nerve fibers have been identified inside the ovary [25]. However, NGF and its receptors are absent in porcine oocytes of primordial and primary follicles but are expressed in the follicular cells of primary follicles. From the secondary stage onward, NGF and its receptors are expressed in oocytes, GCs, and TCs, reaching peak expression in preovulatory follicles and in the corpus luteum (CL) [26,27].

In the human ovary, NGF is expressed in fetal and secondary oocytes and GCs, while TrkA is highly expressed in both GCs and the oocytes of the primary follicles, even if their expression decreases through the antral stage. The other receptor, p75NTR, is detectable in TCs from growing follicles. In antral follicles, NGF is present in both FF and somatic cells, while TrkA is expressed in oocytes [28,29,30]. Notably, NGF concentration in human FF is approximately five times higher than in serum, indicating robust local production [31]. During aging, ovarian functions decrease, along with a decrease in the number of neurons. In senescent rats, neuronal cells are mainly localized in the ovarian stroma and around cysts [32].

## 3. NGF Regulates Follicle and Oocyte Development

The crosstalk between the nervous and reproductive systems is essential for regulating ovarian functions, and many research groups have demonstrated, accross diverse mammalian models, that the activation of the NGF/TrkA axis controls folliculogenesis and the production of fertilizable oocytes [21,33,34].

Experimental results emphasize the role of this signaling pathway in the early stage of follicle development. In mice, genetic or pharmacological blockade of TrkA leads to increased follicular atresia and decreased oogonial survival due to changes in apoptosis-related gene expression [35,36]. By contrast, injecting NGF into the ovaries of NGF-null mice markedly boosts the number of primordial follicles [37] and activates their recruitment through the PI3K/AKT pathway [38]. Additionally, NGF can interact with other regulatory factors, such as TGF-β and GDNF, to ensure coordinated follicular development [21]. In vitro experiments showed that NGF regulates glycolysis and gene expression in mouse CCs as well as the expression of genes involved in the control of oocyte meiotic maturation [39].

In human ovaries, NGF is not essential for follicle assembly, but its absence reduces the pool of primordial follicles and impairs their recruitment in the gonadotropin-dependent development phase [21,22]. NGF increases FSHR expression in GCs, promotes TC proliferation [28,40] while downregulating cyclin-dependent kinase inhibitor CDKN1A (p21) [41]. The increased responsiveness of preovulatory follicles and CCs to gonadotropin stimulation [42] prompts steroidogenesis, and the estrogen-dependent synthesis and release of pro-NGF, whose level rises significantly in FF before being cleaved into the mature form [43]. High concentrations of NGF have also been found in the FF of sheep large antral follicles (>4 mm) in comparison with smaller (<3 mm) or atretic follicles [44]. Human GCs, when stimulated in vitro with NGF, secrete VEGF into the culture medium, but VEGFproduction ceases upon TrkA blockade [45]. As expected, such silencing dramatically reduces follicular cell proliferation by inhibiting the MAPK/ERK pathway [40].

From these results, it is evident that the modulation of MAPK/ERK and PI3K/AKT is central to translating the neurotrophic signal into the follicular microenvironment. MAPK/ERK are usually known to activate phosphorylation and transcriptional factors, such as CREB (cAMP response element-binding protein) and c-FOS/c-JUN, both involved in the regulation of *Cyp19a1* aromatase gene. PI3K/AKT is crucial for the stability and activity of many important transcriptional regulators of ovarian functions [46,47]. Its activation is associated with increased FSHR expression and modulation of FOXL2, both of which are necessary to maintain GC identity and regulate Cyp19a1 expression [48,49].

NGF can mimic gonadotropin effects in cultured sheep cumulus–oocyte complexes (COCs) by promoting cumulus expansion, disrupting gap junction communication through increased connexin 43 phosphorylation [50], and triggering meiotic resumption in the majority (>70%) of oocytes [24,42,44]. More recently, in vitro experiments using mouse COCs showed that NGF regulates glycolysis and the expression of genes involved in the control of oocyte meiotic maturation [39]. NGF contributes to ovulation by stimulating prostaglandin E2 (PGE2) release and by coordinating the complex inflammatory-like response necessary for follicular rupture [51]. Importantly, the ovulatory process is greatly suppressed when anti-NGF antibodies are administered, due to decreased PGE2 release [17] or TrkA receptor deletion [18]. After ovulation, NGF stimulates progesterone production by the ovarian corpus luteum [52].

Surprisingly, NGF may have specie-dependent effects when supplemented during in vitro maturation and fertilization. It has been reported that sheep embryo development is significantly enhanced by its presence [53], while in porcine oocytes, NGF improves maturation efficiency without a corresponding increase in developmental competence [54]. This difference is probably due to different NGF concentrations used in the two studies (100 ng/mL in sheep versus 1 ng/mL in pigs).

**Figure 2:**
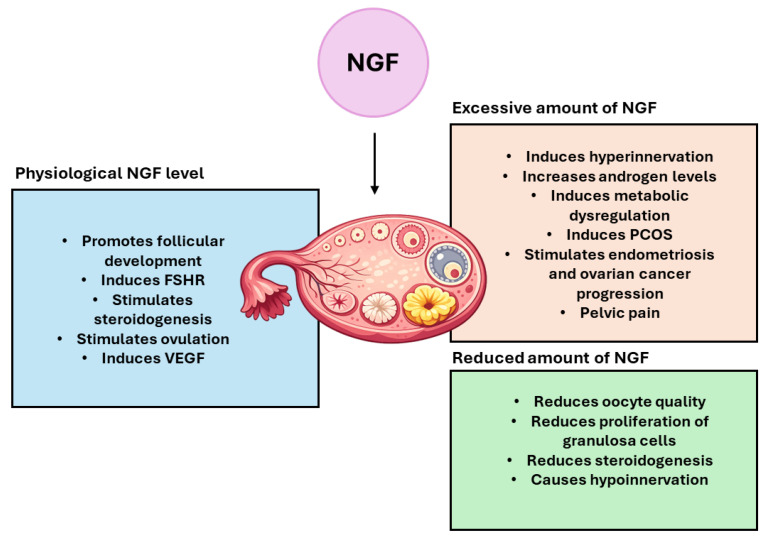
NGF functions in the mammalian ovary. NGF actively participates in the physiological regulation of ovarian cells proliferation and maturation. However, when its levels are altered, NGF causes infertility and other pathological conditions leading to reproductive and metabolic diseases.

These findings indicate that the interaction between NGF and TrkA enables an integrated network in which neurotrophic factors and hormones cooperate to modulate follicular survival, proliferation, metabolism, and development (Figure 2).

## 4. NGF Dysregulation as a Cause of Ovarian Diseases

The complex and multifaceted role of NGF in female fertility is confirmed by the findings that insufficient or excessive levels can adversely affect reproductive outcomes. Since some frequent female reproductive disorders are caused by NGF overexpression, in Table 1, a summary of the effects observed following chronic and acute exposure to high NGF concentrations has been reported.

### 4.1. Infertility

Female infertility is closely linked to poor oocyte quality, which is dependent on hypothalamic dysfunctions, ovulatory disorders, or tubal and uterine abnormalities.

Moreover, the dysregulation of NGF production significantly impacts female fertility, as its overexpression causes ovarian hyperinnervation and follicular dysfunctions. Manti et al. [55] used a mutant mouse model to show that elevated ovarian NGF levels are associated with decreased fertility, altered folliculogenesis, and systemic metabolic problems, including abnormal adipose tissue structure, impaired glucose metabolism, and hepatic steatosis. These ovaries also showed reduced oocyte quality and the abnormal expression of steroidogenic enzymes, including aromatase. Likewise, in animal models, the pharmacological inhibition or genetic deletion of TrkA leads to decreased fertility and defective follicular development [52,61].

In humans, NGF concentrations in FF may correlate with ovarian quality; congruently, infertile women often have low NGF levels in their FF [31], reinforcing its potential as a biomarker for assisted reproductive technologies. Low NGF leads to reduced expression of metabolic genes like *Pfkp* and *Ldha*, which diminish GC’s ability to support oocyte maturation, and of *Gdf9*, *Bmp15*, and *Fgf8*, which help create a suboptimal microenvironment for oocyte development. Conversely, excessive NGF production can result in poor ovarian reserve and hinder meiotic maturation [39].

Physiological levels of NGF are also essential for proper pregnancy progression. Walsh et al. [62] reported that circulating NGF levels stay relatively stable during the first trimester and increase significantly in the later stages of gestation. Elevated NGF levels have been found in women with gestational diabetes or preeclampsia [63], while reduced placental NGF expression has been linked to preterm birth [64]. Frank et al. [65] showed that both excessive NGF level and its neutralization raise the risk of miscarriage, associated with inflammatory responses and immune cell infiltration. Additionally, placental tissues from spontaneous abortions showed significantly higher NGF concentrations compared to full-term controls. In mouse models, increased NGF levels induce lipid peroxidation and increase cyclooxygenase expression in the maternal decidua, thereby promoting preterm labor [66].

Despite these important observations, research on NGF and female infertility remains limited, and more studies are needed to understand its mechanisms and potential therapeutic applications. In contrast, the role of NGF in male fertility is well established, and it is already recommended as a treatment for specific types of infertility [67]. Indeed, NGF proved to be involved in spermatogenesis and to influence Gonadotropin-Releasing Hormone discharge. These effects can be exploited as treatments against hypogonadism and to counteract the side effects of chemotherapies on male reproduction [67].

### 4.2. PCOS

PCOS is a leading cause of female infertility, characterized by chronic anovulation, hyperandrogenism, and abnormal follicular development [68]. FF from women with PCOS contains higher NGF levels compared to healthy controls [69,70]. Consistently, transgenic mice overexpressing the *Ngf* gene exhibit disrupted folliculogenesis and increased apoptosis in antral follicles [22,71]. Similarly, excess NGF impairs fetal development and causes placental dysfunctions in mice [55]. Mannerås et al. [72] reported that acupuncture and physical exercise can influence NGF expression. In a rat model of DHT-induced PCOS, animals treated with low-frequency electroacupuncture and regular exercise for 4–5 weeks showed significantly reduced NGF mRNA levels in adipose tissue, along with the partial recovery of normal ovarian structure compared to untreated PCOS controls. Overall, these findings support the link between NGF dysregulation and PCOS, although the specific molecular mechanisms behind this connection remain unclear.

Animal studies further reveal that the placenta of pregnant rats with PCOS shows significant downregulation of NGF expression, while female F1 offspring exhibit altered expression of key neuronal pathways, consistent with the higher incidence of neurobehavioral disorders reported in daughters of PCOS mothers [73]. Similarly, porcine oocytes exposed to excessive NGF during in vitro maturation demonstrate reduced developmental competence, associated with impaired gap junction communication and a lack of expression of the CC genes involved in glycolysis, thereby recapitulating the key features of PCOS.

In women with PCOS, elevated NGF levels in FF disrupt glycolytic activity in CCs by affecting the expression of key regulatory genes such as *Gdf9*, *Bmp15*, and *Fgf8* [74]. Simultaneously, enzymes involved in testosterone and estrogen production are upregulated, leading to increased GC apoptosis mediated by the NGF-dependent overexpression of TNF-alpha and stathmin (STMN1) [75]. Interestingly, treatments targeting the ovary, such as limited ovarian resection or ovarian drilling, may improve fertility by causing localized ovarian trauma that activates dormant primordial follicles by restricting NGF signaling in the vicinity of the trauma [59,76].

### 4.3. Endometriosis

Endometriosis is a complex inflammatory condition marked by highly variable symptoms and limited treatment options due to the elevated expression of inflammatory and apoptotic markers [77,78], especially in the cortical tissue surrounding endometriotic cysts. NGF appears to play a key role in its pathology, as neurites are frequently observed around endometriotic lesions. Both meta-analyses and in silico studies have confirmed elevated NGF levels in the endometrial tissue and serum of affected patients [79,80]. In a mouse model of endometriosis-associated pain, Zaninelli et al. [81] demonstrated that anti-NGF therapy reduced evoked, spontaneous, and thermal pain, while blocking other neurotrophins (e.g., BDNF) or angiogenic factors (e.g., VEGF) had no effect, highlighting NGF as a key pain mediator. Inflammatory signals may further enhance NGF activity, as treating isolated stromal cells with IL-1β increases TrkA receptor expression [82]. For this reason, both NGF and IL-1β are proposed as potential biomarkers for endometriosis. Sreya et al. [83] measured NGF and IL-1β levels in lesions from 12 patients and observed significant variability (50–173%), within lesions from the same individual, emphasizing the disease’s heterogeneity. Additional studies indicate that NGF levels are higher in deep adenomyotic nodules than in ovarian or peritoneal endometriosis, with perineurial and intraneural invasion observed only in deep lesions [84], suggesting NGF involvement in tissue invasion. These findings are supported by Gori et al. [85], who reported significantly higher NGF expression in deep infiltrating endometriosis compared to the eutopic endometrium.

### 4.4. Ovarian Cancer

Ovarian cancer is a deadly disease characterized by late diagnosis and a poor survival rate. Its most common form is epithelial ovarian cancer (EOC), which accounts for about 90% of all cases. It is characterized by overexpression of NGF that increases angiogenesis by stimulating VEGF production in EOC cells via TrkA. Under physiological conditions, NGF and TrkA are expressed at low levels in ovarian surface epithelial cells, but abnormal FSHR expression, dysregulated gonadotropin secretion, and elevated NGF and TrkA levels characterize EOC [70]. Additionally, global gene expression profiles of EOC human samples have identified 22 altered genes, including the NGF/VEGF signaling, PI3K, AKT2, MAPK, and FOXL-2 and steroid binding. The fact that NGF overexpression increases the Bcl-2/BAX ratio supports its anti-apoptotic effects [86]. In EOC cells, NGF controls the activity of a long list of pro-angiogenic and pro-inflammatory mediators [87], among which are cyclooxygenase 2/prostaglandin E2 (COX-2/PGE2) complex, metalloproteinase domain-containing protein 17 (ADAM17), disintegrin, c-MYC (a member of the proto-oncogene Myc transcription factors family), survivin and β-catenin, all involved in cancer progression and survival. Changes in the subcellular distribution of calreticulin described may mediate an invasive phenotype by dysregulating the transcription of the p53 and MAPK pathways, as observed in breast cancer [70,88]. Interestingly, NGF promotes EOC progression by modulating microRNA expression that is associated with NGF/TRKA activation. Alterations of miR-200 family cluster-17-92, and miR-23b are able to stimulate EOC progression [89]. Vera and collaborators [10] found that the tumoral effect of NGF/TRKA depends on miR-145 regulation in EOC, as NGF decreases f miR-145 transcription, thereby increasing levels of oncogenic proteins involved in tumor progression. Experiments conducted on some EOC cell lines by Garrido et al. [90] revealed that COX-2 inhibition can prevent the NGF-induced increases in COX-2, PGE2, and VEGF levels, suggesting that COX-2/PGE2 signaling inhibition could have an important and beneficial therapeutic role in the treatment of EOC.

### 4.5. Pre-Eclampsia

Pre-eclampsia is a pregnancy-related severe condition based on hypertension that usually shows up before the first 20 weeks of gestation [91]. It is linked to fetal complications and premature birth [92]. Physiologically, trophoblast invasion of the uterine arteries is the initial step in ensuring fetal nutrition during pregnancy [93]. The failure of this step is associated with placental ischemia, hypoxia, and oxidative stress [93]. From a molecular perspective, the release of soluble antiangiogenic factors, such as fms-like tyrosine kinase 1 (sFLT1), has been associated with increased blood pressure [94]. Recently, NGF has been associated with this condition, too. In a longitudinal study, the authors measured NGF levels in pregnant women during gestation and at delivery, comparing levels in maternal and cord blood [63]. While no differences in maternal blood NGF levels were observed, significant differences in cord blood NGF levels were observed between the two groups. In particular, NGF levels were higher in women with pre-eclampsia and newborns’ head diameters were positively associated with NGF concentrations. These results are fundamental introducing the idea that NGF assessment must have clinical relevance [95].

## 5. The Role of Pro-NGF

In this last section, we briefly discussed the role of pro-NGF in the ovary, because it is less studied but potentially crucial. Pro-NGF is not only the precursor of NGF, but also a bioactive molecule with specific functions. Its binding with p75NTR and sortilin can lead to apoptosis and cellular turnover [96,97]. This mechanism appears to be fundamental in tissues where the balance between survival and apoptotic signals is essential, such as in ovarian follicles. Indeed, during follicular development, only a few of them survive while the major part goes through follicular atresia [98]. In this context, the pro-NGF role may counterbalance NGF pro-survival signals. The discussed co-expression of p75NTR and TrkA may support the “switch”hypothesis for these molecules. Pro-NGF activates the JNK pathway and caspases, favoring apoptosis and stress-related responses (Figure 1). To our knowledge, only one paper has studied the role of pro-NGF in GCs. Here, the authors proved that pro-NGF was overexpressed in GCs without a consequent alteration in pro-apoptotic factors. These results prove that pro-NGF is produced by GCs with no involvement in cell death [99]. Additionally, cleavage of the mature NGF may be mediated by MMP7 (matrix metalloproteinase-7), which is also produced by GCs [99]. Considering the contrasting results with what is known about pro-NGF function, combined with the absence of pro-apoptotic signals in this study, further experiments are needed to clarify the specific functions of the NGF precursor and to delineate how the switch from pro-NGF to its mature form can contribute to female health and disease.

## 6. Conclusions

In this review, we analyzed the role of NGF in female health and disease, discussing the experimental results obtained in animal models and humans. From these data, it is concluded that NGF displays an undisputed role in regulating ovarian dynamics and physiology, and dysfunctions in the NGF/TrkA pathway are directly involved in the onset of many common female reproductive diseases. A useful application of NGF that can improve IVF outcomes is its antioxidant action in sperm cryopreservation. However, further studies are needed to better understand the role of this neurotrophinin in the pathophysiology of the male and female reproductive systems.

## Figures and Tables

**Table 1:** Comparison between chronic and acute effects of NGF.

	Chronic, High-Level Exposure to NGF	Acute Injury Induced NGF Expression
Final effects	PCOS; chronic inflammation	Tissue injury, ovulation, transient cellular stress
NGF Levels	Excessively high	Temporarily increased
Innervation	Sympathetic over-innervation of the ovary	Transient neural activation without long term remodeling
Cellular Targets	Theca cells; stroma	Granulosa cells, local stromal cells
Pathways	TrkA-PI3K/AKT-MAPK/ERK	Transient TrkA signaling
Folliculogenesis	Follicular arrest and cyst formation	Follicle early activation
Steroidogenesis	Altered androgen/estrogen balance	Support of estrogen synthesis via Cyp19a1
FSHR and Gonadotropins	Dysregulated	Increased sensitivity of granulosa cells
Ovary	Ovarian dysfunctions; endocrine imbalance	Tissue repair and homeostasis regulation
References	[22,55,56,57,58]	[35,59,60]

## Data Availability

No new data were created or analyzed in this study.
